# Motivational Orientations and Organizational Citizenship Behaviors: The Moderator Role of Perceived Discrimination in the Brexit Context

**DOI:** 10.3390/bs9030031

**Published:** 2019-03-20

**Authors:** Samuel Fernández-Salinero San Martín, Gabriela Topa

**Affiliations:** 1 Medicine and Surgery, Psychology, Preventive Medicine and Public Health and Medical Microbiology and Immunology Department, Universidad Rey Juan Carlos, 28933 Madrid, Spain; 2 Social and Organizational Psychology Department, The National Distance Education University, 28015 Madrid, Spain

**Keywords:** motivational orientations, learning orientation, performance goal orientation, performance-avoid goal orientation, citizenship behavior, organizational identification, perceived discrimination

## Abstract

The current study aims to explain how motivational orientations influence organizational citizenship behavior (OCB) through organizational identification considering the moderator effect of perceived discrimination. A sample of 286 Spanish immigrants in the United Kingdom was included. Main conclusions support that learning orientation shows a significant direct relation with OCB. Both performance and performance-avoid goal orientation have a statistically significant impact on OCB, and this impact is mediated by organizational identification and moderated by perceived discrimination. The practical implications of the study and its possible limitations are discussed.

## 1. Introduction

The exit of the United Kingdom from the European Union, a process designated as Brexit, raises questions for organizations based in the country and for the people involved in them. Brexit promoted a differentiation between the British and other European citizens that is a potential source of perceived discrimination. Thus, hate crimes have increased up to 500% after Brexit consultation, and news about xenophobic waves suffered by immigrants has been disseminated. Moreover, some sectors of the British government have recommended imposing fines on companies that hire European workers [1]. In the same sense, Brexit impacts on the British National Health System (NHS), as the number of foreign nurses who signed employment contracts decreased from 797 in 2015 to 194 in 2017 [2]. Therefore, organizations question whether this new situation can impact on the attitudes and behavior of Europeans working in companies in the United Kingdom [3].

People differ in how they address new situations depending on whether they perceive such situations as challenges or threats. Motivational orientations are essential to understand why some people perceive events as a challenge or a threat. Therefore, organizations are paying attention to motivational orientations due to their possible influence on employee performance [4]. Labor organizations rely increasingly on employee performance to achieve their goals. However, performance is not limited to the mere fulfillment of the tasks described in the job, but is understood in a broad sense, including organizational citizenship behaviors (OCBs). These consist of individual behaviors that are not directly or explicitly recognized by the formal reward system, but that contribute to the effectiveness of the organization. Empirical evidence suggests that motivational orientations could influence OCB [5]. Insofar as the job is challenging, motivational orientations may influence job performance in the sense that they are related to the way of dealing with new situations. In particular, some forms of motivational orientation, such as learning orientation, have been found to favor OCB [6].

Moreover, the way in which people tend to cope with new situations may have a differential impact on the behavior of workers who perceive a discriminatory situation. Recent studies propose that the consideration of future consequences of behavior has significant positive impact on employee performance. These effects are strongest when employees perceive little organizational support [7]. In a moment of crisis and segregation, where ethnic groups are more salient, the perception of discrimination toward immigrants may have an impact on immigrant workers’ OCB. Perceived discrimination runs counter to the clause of dignified treatment that defends the psychological contract, and recent studies show that the attributions of blame for the breach of this contract have a negative impact on OCB [8].

Therefore, this study explores how motivational orientations influence the OCB of migrant workers in the United Kingdom through identification with the organization, taking into account the moderator effect of perceived discrimination. The findings will help to explain the different ways in which people adapt to their organizational environment, and the impact of these differences on their performance. In addition, they will help us to understand how people use their behavior to redefine their status within the group and try to control their situation within the organization. Finally, they may help to design intervention models aimed at encouraging a greater number of OCBs through the promotion of some motivational orientations.

### 1.1. Motivational Orientations as Predictors of OCB

Motivational orientations can be defined as the individual’s guide for the interpretation of knowledge and the production of cognitions, emotions, and behaviors when facing tasks [9]. The literature has pointed out the existence of two types of motivational orientations, performance orientations and learning orientations, which have been identified as two distinct dimensions [8]. Learning orientation is characterized by the search for perfection in new situations and the acquisition of skills. Performance orientation is related to the attempt to demonstrate and validate one’s skills and competencies to others, trying to achieve positive judgments and avoid negative ones [10].

Performance orientation can, in turn, be seen as a two-dimensional construct: performance goal orientation and performance-avoid goal orientation. Performance goal motivation refers to individuals’ efforts compared with their own standards of excellence. Performance-avoid goal motivation is based on the desire to avoid negative judgments about one’s competencies. Therefore, this predisposes one toward anxious evaluation and sensitivity toward failure that biases the information [11]. People with a predominance of performance goal orientation will tend to try harder and to prove that they can excel themselves. People with a predominance of performance-avoid goal orientation will try not to embark on activities that may receive negative feedback, tending to respond with minimal effort [12].

Each person, as a function of their motivational orientation, holds implicit theories about the controllability of their personal attributes, such as intelligence. These theories predispose people to different goal orientations [13]. Performance-oriented subjects rate their skill as a fixed and uncontrollable quality. In contrast, individuals focused on learning see that their capabilities can vary due to practice and effort.

Recent studies [14] have shown that job insecurity is a potential job stress factor. When a person perceives that is a discrimination object, he may feel more vulnerable and perceive distress as the Social Identity Theory shows. Stress can be faced in a behavioral way through self-affirmation which may be related to learning orientation, or work-affirmation which may be related to goal orientation. Taking into account the foregoing consideration, both orientations influence the way in which people perceive their own efforts. On the one hand, with a focus on learning, effort is seen as an investment for the development of skills. This response pattern is adaptive and leads to better results. On the other hand, in performance orientation, effort is seen as a sign of little skill because a talented person would not need to strive; therefore, it leads to a maladaptive pattern in challenging situations. Hence, since OCBs are extra-role behaviors, we propose that different motivational orientations could influence OCBs, as outlined above.

Therefore, it seems appropriate to investigate possible significant differences in OCBs on the basis of these motivational orientations in a discriminatory environment.

### 1.2. Organizational Identification

People can be defined through their individual identity and by belonging to different groups; that is, through their social identity. First of all, with the term organization we refer to a business, understood as an entity aimed at carrying on commercial enterprise by providing goods or services, to meet needs of the customers or users. Identification with the organization is a form of social identity through which the person feels psychologically joined to the group. As social identification is the part of the conception of the self that derives from belonging to different social groups, it is linked to emotional and evaluative meanings attached to that membership [15]. Thus, group membership serves to define oneself in the social environment and also determines the behavior that one is willing to perform as a member of that group. Organizational identity “turns on” pro-organizational behavior.

In addition, it seems clearly established that, as people become more strongly identified, they are more likely to act in accordance with a social identity that is implied by membership in a group [16].

Thus, identification is associated with many desirable outcomes in organizations, such as extra-role behavior, contextual performance, and OCB [17]. Therefore, organizational identification has been studied as a mediator in a wide range of organizational behaviors, such as leadership, job satisfaction, and entrepreneurial behavior, among others. On the basis of this evidence, the present study aims to explore the mediator role of organizational identification in the relationship between motivational orientations and OCB.

Organizational identification may act as a nexus between the subject and the organization. As the social identity theory framework states, people make efforts to feel part of a group. We assume that people who identify more strongly with the organization will be more motivated to become a desirable member of the group, acting accordingly to their social identity.

In particular, a higher learning orientation is expected to promote organizational identification, and high levels of identification may create a fertile ground for OCB (Hypothesis H1a). In the same sense, a higher performance goal orientation is expected to promote organizational identification, and high levels of identification may create a fertile ground for OCB (Hypothesis H1ba). Finally, a higher performance-avoid goal orientation is expected to harm organizational identification, whereas lower levels of identification may create an impediment for OCB (Hypothesis H1c).

### 1.3. Perceived Discrimination

Stigmatized group membership can be a highly stressful phenomenon. Belonging to stigmatized groups inevitably entails personal dissatisfaction, self-rejection, and low self-esteem. In addition, perceived prejudice related to a person’s global and stable categories such as nationality or skin color does more harm to well-being than perceived prejudice toward some particular feature [18]. How one copes with the relationship with other groups and other people, and the personal value the individual attributes to membership are key factors in these relationships.

In the Brexit setting, nationality-related social identity becomes salient. Spaniards who emigrated to the United Kingdom may have conflicts when perceiving their ingroup as stigmatized, and this perception can affect their performance. Therefore, these prejudices took place whether the threats were factual or symbolic, in line with intergroup threat theory [19]. Nationality is a stable characteristic; it is assumed that the perception of discrimination due to this factor will impact on how one relates to the organization and how one behaves, affecting OCB. Thus, the tacit rejection of a person because of his or her nationality can generate a climate of stress that affects organizational identification and performance. The perception of the discrimination will moderate the relationship between motivational orientations and OCB, mediated through organizational identification.

Social changes and psychological experience bear differential influences in the motivational implications of potentially activated categorizations, as recent studies show [20]. Our investigation in this direction attempts to shed light on dispositional variables by which the above-mentioned differential influence can be explained. Thus, it may be assumed that strong perceived discrimination could increase the positive impact of a high learning orientation on OCB (Hypothesis H2a). In the same sense, we expected that strong perceived discrimination could increase the positive impact of high performance goal orientation on OCB (Hypothesis H2b). On the contrary, it may be assumed that a strong perceived discrimination could increase the negative impact of a high performance-avoid goal orientation on OCB (Hypothesis H2c).

Within the social identity theory framework, we can suppose that people who are associated with a stigmatized group will make efforts in order to redefine their membership. So, we suppose that there will be differences in coping with this stressful membership inside the organization through OCB. Moreover, we can suppose that the coping responses will vary depending on the personal motivational orientations. In summary, perceived discrimination, being a potential harmful event for identity, will moderate the relationship between motivational orientations and OCB. This is because being a useful and desirable member of a group is a central motivation within the social identity framework.

In sum, the current study aimed to assess whether the relation between motivational orientations and OCB was mediated by a higher perception of organization identity, and whether the effect of motivational orientations on organizational identification was moderated by perceived discrimination.

## 2. Materials and Methods

### 2.1. Participants

The sample was made up of 286 subjects; all of them were Spanish citizens residing and working in the United Kingdom, and 68.9% (197) were men and 31.1% (89) were women. Mean age was 29.62 years (SD = 6.40). Concerning educational level, 71.3% of the sample comprised people with university education, compared to 18.5% who reported professional training, 9.1% with secondary school or high school, and 1% with primary or basic studies. Regarding working conditions, 78.3% of the sample had a permanent contract versus 21.7% who were hired on a temporary basis.

In addition, 79.4% worked full-time, and 20.6% worked part-time. Average tenure in the organization was 17.3 months (SD = 22.2). In relation to permanence in the United Kingdom, 36.4% of the sample had lived there less than six months, 27.9% between 7 months and one year, and 35.7% more than one year. Lastly, 60.6% of the sample lived in London. The migrant workers in the United Kingdom who participated in the study came from Andalusia (23.8%) and Madrid (21.6%).

### 2.2. Procedure

To access the potential participants in the study, we contacted the administrators of Facebook of Spanish citizens residing in England groups. The possibility of participating in the study was published, and we sent the questionnaire via e-mail to the people who wished to participate. Potential participants were informed about the objective of the study, the conditions of anonymity and confidentiality of the data, and they answered a few questions expressing their informed consent. This study was approved by the committee of ethics of The National Distance Education University , dated October 2016.

### 2.3. Instruments

The measures exceeded the reliability criterion of Nunnally, with Cronbach alpha indices higher than 0.70.

#### 2.3.1. Motivational Orientations

We used the motivational orientation inventory [21] to assess this variable. Specifically, the subscale of mastery of goal-oriented work was translated for this research. It assesses three dimensions: (a) Learning orientation through 5 items (“I often look for opportunities to develop new skills and knowledge”), (b) Performance goal orientation through 4 items (“I enjoy it when others at work are aware of how well I’m doing”), and (c) Performance-avoid goal orientation through 4 items (“I would avoid taking on a new task if there is a chance that I might appear rather incompetent to others”). The response format is a 5-point Likert-type scale (1 = strongly disagree, 5 = strongly agree). The original inventory obtained reliability indices (Cronbach alpha) of 0.85, 0.84, and 0.80, respectively.

#### 2.3.2. Organizational Identity

We used the organizational identity questionnaire of Mael and Ashforth [22], which has been widely used in the organizational sphere (“When someone criticizes the company where I work, I take it personally”; “I am very interested in what others think of the company where I work”). The responses of the 6 items are rated on a 5-point Likert-type scale (1 = strongly disagree, 5 = strongly agree). The original instrument yields reliability values (Cronbach alpha) over 0.80.

#### 2.3.3. Perceived Discrimination

The identity-ethnic minority experience questionnaire [23] is composed of two subscales. The first subscale (a) is made up of two items that refer to the status of the ethnic group in question (“What opinion do you think the English have of Spanish immigrants?”). The second subscale (b) refers to the perception of discrimination and social exclusion and is made up of six items (“Have you ever been treated rudely by the English and without considering your feelings?”). Responses are rated on a Likert-type scale (range of 1 = Never to 5 = Always). The first subscale is not included in the questionnaire, following the precedent of the authors in the study cited in this paragraph. The reliability (Cronbach alpha) was 0.79.

#### 2.3.4. Organizational Citizenship Behaviors

To measure this variable, we used the Spanish validation of the Organizational Citizenship Behavior Organization (OCBI/OCBO) scale [24] carried out by Davila and Finkelstein [25]. We used the OCBO subscale (Organizational Citizenship Behavior Organization—Organization-focused OCB). It consists of eight items, scored on a Likert-type scale (1 = never, 5 = always) about the OCB towards the organization as a whole (“I attend to functions that are not required, but that promote the image of the organization”; “I am proud when I represent the organization in public”). The decision not to use the OCBI (person-oriented OCB) scale was taken due to previous studies [26]. It was shown that OCBI were not important for the prediction of organizational variables, such as job satisfaction, and the OCBO scale was more efficient. The reliability (Cronbach alpha) of the original global instrument was 0.81 [25].

## 3. Results

The data were analyzed using SPSS v.24, with regression analysis, using the PROCESS macro wich is an observed variable OLS and logistic regression path analysis modeling tool for SPSS and SAS. As suggested by Hayes [27], before testing the hypothesized moderated mediation model, the indirect and moderating effects were first tested separately. The simple mediating effect analysis examines how the predictor affects the criterion variable, whereas the simple moderating effect analysis shows when the predictor may influence the criterion variable. Subsequently, a moderated mediation model estimating all parameters simultaneously was tested using the PROCESS macro for SPSS [26]. In particular, the hypothesized relationships were assessed using Model 7, which estimates the indirect effect of X (motivational orientation) on Y (OCB) through M (organizational identification), with a moderating role played by W (perceived discrimination) in the X→M (motivational orientation→T1 organizational identification) relationship. The moderated mediation hypothesis is supported when the mediation process varies at different values assumed by the moderating variable. This procedure was based on 1000 bootstrap re-samples and provided an index of moderated mediation, as well as estimates of the indirect effect and associated confidence intervals conditional on specific levels of the moderator (Mean and ± 1 SD from Mean). When zero is not included in the 95% bias-corrected confidence interval, it may be concluded that the parameter is significantly different from zero at *p* < 0.05. Moreover, gender and organizational tenure were included as covariates.

Before testing our model, a correlation analysis was conducted among the study variables. These results are reported in Table 1. Pearson’s correlations indicated that all significant relationships between the variables were in the expected direction.

### 3.1. Simple Mediation Analysis

The first analysis examined the indirect effect of motivational orientations on OCB through organizational identification. Firstly, the results indicated a positive effect of learning orientation on organizational identification (B = 0.66, SE = 0.25, 95% CI [0.17, 1.15], *p* = 0.008), and a positive association between organizational identification and OCB (B = 0.42, SE = 0.04, 95% CI [0.34, 0.51], *p* < 0.001). These results supported the significance of the direct effect of learning orientation on OCB (B = 0.23, SE = 0.05, 95% CI [0.12, 0.35], *p* = 0.000) and the indirect effect between these variables (B = 0.10, SE = 0.03, 95% CI [0.04, 0.17]), mediated by organizational identification. Subsequent Sobel tests supported this result (z = 2.78, *p* = 0.005). In our study, age and job tenure were not significantly related to the perception of organizational identification and OCB. Taken together, these results indicated a significant mediating effect of organizational identification in the relationship between learning orientation and OCB.

Secondly, the results indicated a negative effect of performance goal orientation on organizational identification (B = −0.26, SE = 0.19, 95% CI [−0.62, 0.11], *p* = 0.17), and a positive association between organizational identification and OCB (B = 0.43, SE = 0.04, 95% CI [0.35, 0.51], *p* < 0.001). These results supported the significance of the direct effect of performance goal orientation on OCB (B = 0.12, SE = 0.04, 95% CI [0.04, 0.20], *p* = 0.005) and the indirect effect between these variables (B = 0.08, SE = 0.03, 95% CI [0.031, 0.14]), mediated by organizational identification. Subsequent Sobel tests supported this result (z = 2.98, *p* = 0.003). In our study, age and job tenure were not significantly related to the perception of organizational identification and OCB. Taken together, these results indicated a significant mediating effect of organizational identification in the relationship between performance goal orientation and OCB.

Thirdly, the results indicated a negative effect of performance-avoid goal orientation on organizational identification (B = −0.47, SE = 0.17, 95% CI [−0.80, −0.13], *p* = 0.007), and a positive association between organizational identification and OCB (B = 0.45, SE = 0.05, 95% CI [0.36, 0.53], *p* < 0.001). These results supported the significance of the direct negative effect of performance-avoid goal orientation on OCB (B = −0.11, SE = 0.04, 95% CI [−0.18, −0.03], *p* = 0.005). But the indirect effect between these variables (B = −0.01, SE = 0.03, 95% CI [−0.062, 0.04]), mediated by organizational identification, was not significant. Subsequent Sobel tests supported this result (z = −0.481, *p* = 0.63). In our study, age and job tenure were not significantly related to the perception of organizational identification and OCB. Taken together, these results indicated a significant mediating effect of organizational identification in the relationship between performance-avoid goal orientation and OCB.

### 3.2. Moderation Analysis

The second analysis explored the moderating effect of perceived discrimination on the association between motivational orientations and organizational identification. Firstly, related to the learning orientation, the overall model was significant, F (3, 282) = 4.63, *p* = 0.004, R2 = 0.05. The main effect of learning orientation (B = 0.66, SE = 0.25, 95% CI [0.17, 1.15], *p* = 0.008) was significant, while the effect of perceived discrimination (B = 1.13, SE = 0.72, 95% CI [−0.28, 2.53], *p* = 0.12) was not significant, as was the interaction term (B =−0.29, SE = 0.16, *p* = 0.07, ΔR2 = 0.02).

**Figure 1:**
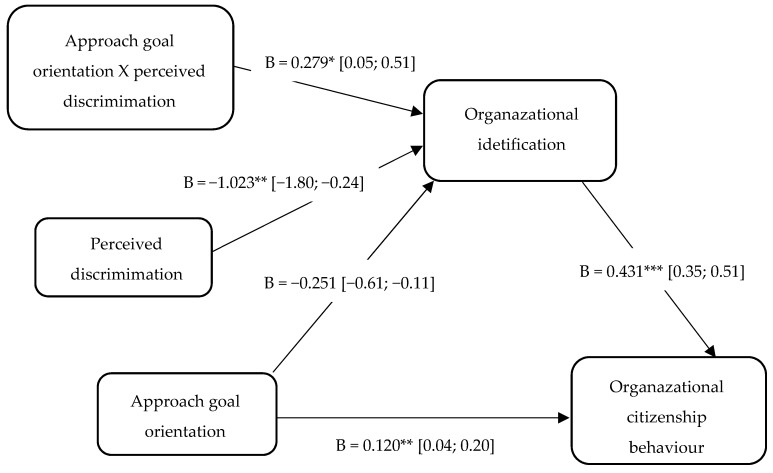
Results of the moderated mediation analysis. The relationship between approach goal orientation and organizational citizenship behavior (OCB) through organizational identification was moderated by perceived discrimination. Note: * *p* < 0.05. ** *p* < 0.01. *** *p* < 0.001.

Secondly, related to the performance goal orientation, the overall model was significant, F (3, 282) = 5.57, *p* = 0.0014, R2 = 0.06. The main effects of the performance goal orientation (B = −0.26, SE = 0.18, 95% CI [−0.62, 0.11], *p* = 0.17), were not significant, while the effect of perceived discrimination (B = −1.02, SE = 0.39, 95% CI [−1.8, −0.24], *p* < 0.01) was significant, as was the interaction term (B = 0.28, SE = 0.12, *p* = 0.02, ΔR2= 0.02). Specifically, the results indicated that the association between performance goal orientation on organizational identification decreased in magnitude from high (−1SD; B = 0.31, SE = 0.08, *p* < 0.001) to low (+1SD; B = 0.03, SE = 0.08, *p* = 0.73) levels of perceived discrimination. Consistent with our expectations, employees reporting a higher performance goal orientation perceive a lower level of organizational identification if they have a strong perception of discrimination (Figure 1).

**Figure 2:**
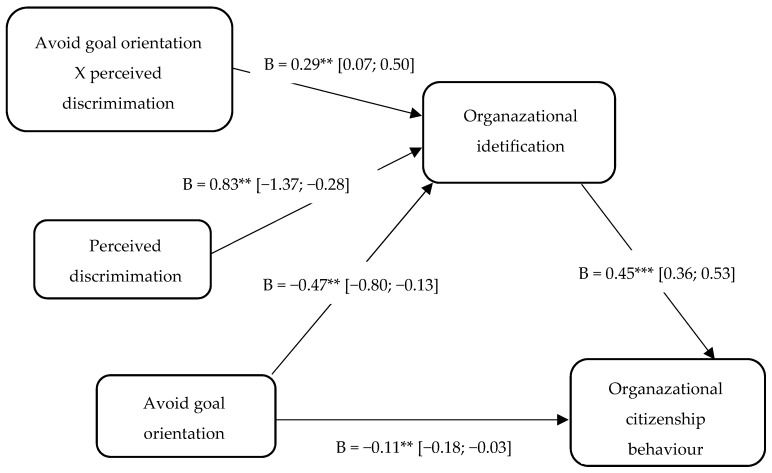
Results of the moderated mediation analysis. The relationship between performance-avoid goal orientation and OCB through organizational identification was moderated by perceived discrimination. Note: * *p* < 0.05. ** *p* < 0.01. *** *p* < 0.001.

Thirdly, related to the performance-avoid goal orientation, the overall model was significant, F (3, 282) = 3.10, *p* = 0.02, R2 = 0.03. The main effects of both performance-avoid goal orientation (B = −0.47, SE = 0.17, 95% CI [−0.80, −0.13], *p* = 0.007) and the effect of perceived discrimination (B = −0.83, SE = 0.27, 95% CI [−1.36, −0.28], *p* < 0.01) were also significant, as was the interaction term (B = 0.29, SE = 0.11, *p* < 0.01, ΔR2 = 0.03). Specifically, the results indicated that the association between performance-avoid goal orientation on organizational identification was negative and significant when perceived discrimination was high (−1SD; B = −0.17, SE = 0.07, *p* < 0.05) and was positive and lost its significance when perceived discrimination was low (+1SD; B = 0.11, SE = 0.07, *p* = 0.13). Consistent with our expectations, employees reporting a higher performance-avoid goal orientation perceive a greater and negative level of organizational identification if they have a strong perception of discrimination (Figure 2).

### 3.3. Moderated Mediation

Firstly, for the learning orientation dimension, the index of moderated mediation was not significant (B = −0.12, SE = 0.07, 95% CI [−0.27, 0.006]). Moreover, this analysis revealed a conditional indirect effect of learning orientation on OCB through organizational identification, with the indirect effect being not significant at low levels of perceived discrimination (−1SD; B = 0.15, SE = 0.04, 95% CI [0.07, 0.26]) or at high levels of perceived discrimination (+1SD; B = 0.03, SE = 0.05, 95% CI [−0.07, 0.13]). Secondly, for the performance goal orientation dimension, the index of moderated mediation was significant (B = 0.12, SE = 0.05, 95% CI [0.02, 0.22]). Moreover, this analysis revealed a conditional indirect effect of the performance goal orientation on OCB through organizational identification, with the indirect effect being insignificant at low levels of perceived discrimination (−1SD; B = 0.02, SE = 0.04, 95% CI [−0.06, 0.09]), but significant at high levels of perceived discrimination (+1SD; B = 0.13, SE = 0.03, 95% CI [0.07, 0.19]). Thirdly, for the performance-avoid goal orientation dimension, the index of moderated mediation was significant (B = 0.13, SE = 0.05, 95% CI [0.04, 0.24]). Moreover, this analysis revealed a conditional indirect effect of performance-avoid goal orientation on OCB through organizational identification, with the indirect effect being significant at low levels of perceived discrimination (−1SD; B = −0.08, SE = 0.04, 95% CI [−0.15, −0.02]), while this indirect effect was not significant at high levels of perceived discrimination (+1SD; B = 0.05, SE = 0.04, 95% CI [−0.02, 0.13]).

## 4. Discussion

The present study explores the effect produced by motivational orientations on OCB, mediated by organizational identification, taking into account the moderator effects of perceived discrimination within the context of Brexit. We took into account the limitations of previous research, which posited that either there were no statistically significant differences between performance orientations and desirable behaviors, or the performance-avoid goal orientation was associated with lower performance levels [28]. From the perspective of this study, we proposed a fundamental difference in the way the two motivational orientations function, as the contextual factor has an important impact on the relationship of the two performance orientations.

In relation to the behavior of people in organizations, this study provides further evidence to previous findings about the relationship between the learning orientation and individual performance [29]. This work proposes a possible explanation of how performance orientations may also be related to OCB. Our study also confirms the evidence about the undeniable influence of organizational identification on OCB [30].

Although it may not occur strictly in the organizational setting, the perception of discrimination in the environment influences how people relate to their organization. It has been shown that individuals who are more susceptible to the judgments of others are more vulnerable to these signs of prejudice. The performance-avoid goal orientation was shown to be positively related to burnout, whereas the performance goal orientation was negatively associated with the syndrome [31]. However, this previous study was carried out in school population and contextual factors, such as the dimension of perceived discrimination, were not taken into account as we propose herein. With regard to the learning orientation, this could act as a protection factor that buffers the adverse effects of perceived discrimination, and it is a possible antecedent of workers’ health. This statement is in the line with the proposals of Niiya, Crocker, and Bartmess [32], who postulated that the learning orientation was an effective way to minimize students’ threats to self-esteem.

Also, people who are performance-goal oriented are more motivated to carry out extra-role behavior. This may be due to the fact that, when perceiving discrimination, these individuals make a greater effort to achieve a positive social identity and seem valid to others. When perceiving discrimination and engaging in OCB, they may redefine their membership in the stigmatized group through processes of social change.

It should be acknowledged that the results related to performance-avoid goal orientation did not achieve the expected significance levels in the present study. However, the findings suggest a trend that when performance-avoid goal orientated people do not perceive discrimination, they prefer to avoid challenges which are not included in the formal contract and thus preserve their positive social identity, avoiding possible negative judgments. In addition, these individuals may show an anxious pattern which could also lead to burnout. In subsequent studies, it would be interesting to test this assertion.

Finally, to the extent that the studies being carried out at present seem to confirm that the favorable vote of Brexit is associated with predictors of prejudice toward foreigners, such as a British collective narcissism, right wing authoritarianism, and social dominance orientation, it seems interesting to develop programs to prevent the increase of collective discrimination and of negative behaviors toward workers from Europe.

It must be acknowledged that this study has multiple limitations. First, the sample is not representative of the population of Spanish immigrant workers in the United Kingdom, nor was it obtained by random sampling procedures. Among other sociodemographic characteristics, it can be observed that the participants are mostly university professionals. This sample bias could impact the findings of this study. Moreover, the effect sizes of the regression coefficients are low. Due to this, the interpretation of these results has to be considered carefully and, if possible, be tested in future research in order to test through external validity.

In addition, the criterion variable of the present study is self-reported and may be subject to the influence of social desirability. A line of future work could be developed through the inclusion of some kind of objective measure, such as absenteeism, or hetero-reported contextual performance measures, following the procedure used in other studies. Another limitation refers to the measurement of perceived discrimination, which is not specific to the Brexit scenario. It would be interesting to develop a specific instrument that evaluates the perception of discrimination since the adoption of such a measure.

This work could be expanded by including other variables, such as the perception of work-related stress or thinking about returning to the country of origin in the future, because these variables may have an impact by moderating the influence of motivational orientations on the criterion variables.

Moreover, although the study yields a noteworthy conclusion, it is also true that some models do not achieve sufficient evidence. In the first place, performance-avoid goal orientation has no statistically significant impact on OCB when it is mediated by organizational identification. Potentially stressful contextual factors (such as the perception of exclusion) for identity are required for them to affect the criterion variable, through moderation of the organization identification process. Hence, when individuals who are motivated to avoid a negative performance undergo a normal identification process, they do not perform fewer OCBs.

Finally, we clarify that, with regard to learning orientation, the moderate mediation model is not significant in the prediction of OCB. The effect of this variable on the criterion is relatively independent of the mediator and moderator variable, as it acts as a buffer of stressors and protects the person’s identity from possible threats arising from failure.

Taking the above into account, a possible avenue of future research would be to assess the levels of burnout according to motivational orientations. In this sense, if a person perceives that not performing OCB provokes a negative evaluation, this can be a source of further stress that leads the employee to a situation of overexertion. Another proposal for future research is to assess the awareness of their professional skills in employees with different motivational orientations. In this regard, some recent research [33] proposes that the workmates of people with performance-avoid goal orientation have a worse perception of these people’s intellectual skills, social qualities, and popularity, which can influence the organizational climate and self-knowledge.

Moreover, it would be of interest to delve into whether motivational orientations are stable over time or vary on the basis of life experience. One last suggestion is to carry out experimental studies to test differential ways of behaving in people with different goal orientations. Investigating possible significant differences in the quality of the work of people with different motivational orientations, taking into account the mediating and moderating variables proposed in this study, could be of interest.

To conclude, in order to achieve socially responsible and health-oriented organizations, it is essential to know the processes and dynamics that come into play within them. This study opens an important avenue for this purpose.

Our study contributes to increase the corpus of present literature about motivational orientations, and extends their implementation, considering contextual variables that should not be neglected when acting in an organizational environment.

The purpose of this research is potentially practical, as it would be interesting to be able to develop human resources programs aimed at promoting a learning orientation, and to help find these profiles in the processes of personnel selection, taking motivational orientations into account when designing jobs or allocating different employees. Our study is in line with current research which shows that the need for competence and relatedness within self determination theory (SDT) bears a significant relation to working environment desirable variables, as well as to the job satisfaction level and psychological well-being of employees, and fewer health problems [34].

On the other hand, it proposes the need to consider organizational identification as a variable through which motivational performance orientations are related to OCB. Finally, the implications for politicians are clear, as the management of exclusionary policies has a direct or indirect impact on performance and, thus, on the economy of the country.

## Figures and Tables

**Table 1:** Descriptive statistics and correlation matrix (*N* = 286).

Variables	*M*	*SD*	α	1	2	3	5	6	7
1. Age (years)	29.6	6.4							
2. Organizational tenure (months)	17.3	22.2		0.21 **					
3. Learning orientation	4.38	0.60	0.84	−0.04	−0.05	−			
4. Prove goal orientation	3.31	0.85	0.69	−0.05	−0.08	0.18 **			
5. Performance-avoid goal orientation	2.41	0.92	0.82	0.09	−0.02	−0.28 **	0.34 **		
6. Organizational citizenship behavior (OCB)	3.57	0.71	0.87	−0.03	−0.07	0.28 **	0.24 **	−0.15 **	
7. Organizational identification	3.20	0.84	0.82	−0.02	−0.07	0.17 **	0.18 **	−0.03	0.54 **
8. Perceived discrimination	1.81	0.81	0.87	0.06	0.02	−0.01	−0.05	0.01	−0.02

*Note*: * *p* < 0.05. ** *p* < 0.01.
